# A Minimally-Invasive Method for Serial Cerebrospinal Fluid Collection and Injection in Rodents with High Survival Rates

**DOI:** 10.3390/biomedicines11061609

**Published:** 2023-06-01

**Authors:** Jingrong Regina Han, Yu Yang, Tianshu William Wu, Tao-Tao Shi, Wenlu Li, Yilong Zou

**Affiliations:** 1 School of Life Sciences, Fudan University, Shanghai 200438, China; 2 Westlake Four-Dimensional Dynamic Metabolomics (Meta4D) Laboratory, Westlake Laboratory of Life Sciences and Biomedicine, Hangzhou 310024, China; 3 School of Life Sciences, Westlake University, Hangzhou 310024, China; 4 Westlake Institute for Advanced Study, Hangzhou 310024, China; 5 Research Center for Industries of the Future, Westlake University, Hangzhou 310024, China; 6 Laboratory Animal Resources Center, Westlake University, Hangzhou 310024, China; 7 College of Life Sciences, Zhejiang University, Hangzhou 310027, China; 8 Neuroprotection Research Laboratories, Departments of Radiology and Neurology, Massachusetts General Hospital, Harvard Medical School, Boston, MA 02129, USA

**Keywords:** cerebrospinal fluid (CSF), central nervous system (CNS) diseases, mouse models, CSF biomarker, CSF collection, CSF injection

## Abstract

Cerebrospinal fluid (CSF) is an important sample source for diagnosing diseases in the central nervous system (CNS), but collecting and injecting CSF in small animals is technically challenging and often results in high mortality rates. Here, we present a cost-effective and efficient method for accessing the CSF in live rodents for fluid collection and infusion purposes. The key element of this protocol is a metal needle tool bent at a unique angle and length, allowing the successful access of the CSF through the foramen magnum. With this method, we can collect 5–10 µL of the CSF from mice and 70–100 µL from rats for downstream analyses, including mass spectrometry. Moreover, our minimally-invasive procedure enables iterative CSF collection from the same animal every few days, representing a significant improvement over prior protocols. Additionally, our method can be used to inject solutions into mice cisterna magna with high success rates and high postoperative recovery rates. In summary, we provide an efficient and minimally-invasive protocol for collecting and infusing reagents into the CSF in live rodents. We envision this protocol will facilitate biomarker discovery and drug development for diseases in the central nervous system.

## 1. Introduction

Diagnosing central nervous system (CNS) diseases is an ongoing challenge in biomedicine. Molecular profiling of the cerebrospinal fluid (CSF) is a common diagnostic approach for diseases of the central nervous system (CNS), including infectious diseases, autoimmune disorders, brain hemorrhage and traumatic brain injury, CNS tumors, and Alzheimer’s disease [1,2,3,4,5,6,7,8,9,10]. The CSF provides an important window and sample source for discovering potential biomarkers to indicate the pathophysiological states of the CNS. Under physiological conditions, the CSF is predominantly secreted from the choroid plexus within the ventricular interior of the brain to circulate in the ventricles and subarachnoid space of the brain and spinal cord [11]. The CSF supports CNS functions by supplying nutrients, removing waste, transduction of signals, and protecting against pathogens [11,12]. The CSF also serves as a cushion to protect the brain and spinal cord tissues from acute mechanical impact-triggered injury.

Due to the pleiotropic functions of the CSF, the compositional analysis of the CSF via multi-omics or targeted approaches is widely used to diagnose and prognose CNS disorders [13]. In addition to the diagnostic value, recent research revealed that infusing the CSF of young mice into aged mice could restore memory [14], highlighting the therapeutic potential of the CSF as a modulatory agent in treating CNS-related diseases. Nevertheless, extracting the CSF from small laboratory animals, including mice, remains technically challenging due to their small body size and limited CSF volume.

Currently, there are three typical routes for CSF collection in mice, including accessing the liquid phase through the cisterna magna, the lateral ventricle and the intrathecal route [15,16,17]. The existing cisterna magna collection method requires a fine and firm puncture by the operator and is incompatible with repeated CSF collection. Likewise, the lateral ventricle collection method requires using a thin metal needle to penetrate the brain parenchyma for stereotaxic targeting towards the ventricle space, which can be inaccurate and may cause brain injury. However, intrathecal cannulation may cause injury to the brain parenchymal tissue due to insertion of the cannula. Collectively, the existing CSF collection methods require both sophisticated instruments and well-trained operators, and could lead to high mortality rates or cause significant distress that reaches euthanization endpoints for animal welfare [15,16,17]. These limitations prevent the exploration of CNS disorders and longitudinal studies that require consecutive sampling of the CSF from the same animal. Thus, an efficient, sustainable and humane CSF collection method is urgently needed.

In addition to assisting in CNS disease biomarker discovery, the CSF also represents an important infusion site for delivering perturbational agents into the CNS. By bypassing the blood-brain barrier, plasmids, adenoviruses, proteins, small molecule drugs and cells injected into the CSF can efficiently reach the choroid plexus cell layer, brain parenchyma and spinal cord tissues [18,19,20,21,22]. Nonetheless, CSF injection in rodents remains a technically challenging task. For instance, the infusion methods for mice CSF access typically require complex surgical operations or rely on mechanical pumps to control the rates of infusion towards the lateral ventricle or intrathecal space [18,19,23].

To circumvent these bottlenecks, here we introduce the bent needle method, which can significantly simplify accessing the mouse and rat CSF space for both fluid extraction and infusion, while ensuring high postoperative survival rates. Considering the vast number of transgenic rodent models and their widespread utility in modeling CNS diseases, we envision that the more streamlined CSF injection methods in small animal models would be useful for many directions of neuroscience research.

## 2. Materials and Methods

### 2.1. Animals

All animal experiments and procedures were conducted in strict compliance with institutional guidelines for animal welfare. The Institutional Animal Care and Use Committee (IACUC) of Westlake University, Hangzhou, China approved all animal studies described in this report. The animal models used in this study consisted of C3H/HeJ mice, C57BL/6 mice and Sprague–Dawley (SD) rats. C3H/HeJ mice aged 8–9 months old were kindly provided by the Laboratory Animal Resources Center of Westlake University. C57BL/6 mice aged between 6–7 months old were purchased from the Laboratory Animal Resources Center of Westlake University. Sprague–Dawley (SD) rats aged 6 months old were provided by Charles River Laboratories, Zhejiang, China. All the animals were housed in 12-h light-dark cycles in an animal facility with regular food and water supplies. All the experiments were conducted inside the animal facility, and upon completion of the experiments, the animals were returned to their individual cages for monitoring.

### 2.2. Surgical Tools and Preparation Processes

#### 2.2.1. Surgical Table

**Figure 1:**
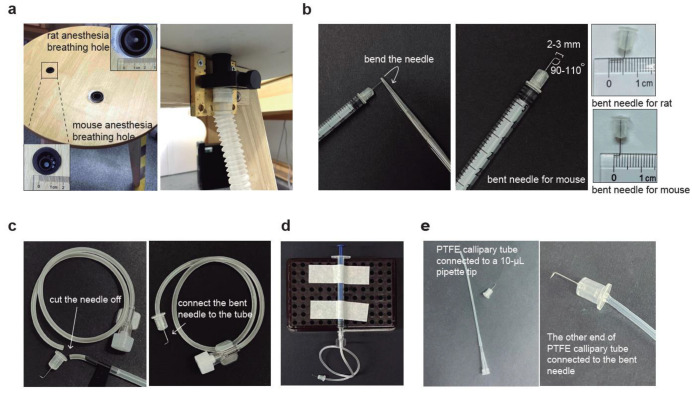
Graphic schemes for the construction of CSF collection and injection tools. (**a**) Custom-made surgical table containing two breathable holes of different sizes for hosting mice and rats, respectively. The two holes are connected to the anesthesia machine with gas tubes. (**b**) The preparation process of a bent needle consists of using forceps to hold the needle at a certain distance and then applying force to bend it. We recommend bending the needle at an angle of 90–110°. The tip length is 2–3 mm and 4–5 mm for mice and rats, respectively. (**c**) Preparation process of surgical needle set by cutting off the needle from the intravenous infusion needle, then connecting the bent needle to the soft tube. (**d**) An overview of the surgical tools for CSF collection by connecting custom-made surgical needle sets to a needleless 1-mL syringe. (**e**) Graphical scheme for the injection tools by connecting the bent needle with the PTFE capillary tube. Once the bent needle end is connected, connect the other end of the PTFE tube to a 10-µL pipette tip and a pipette.

The panel of the surgical wood table was custom-made by drilling holes with various diameters to fit the breathing connection part of the inhaled gas anesthesia equipment (Figure 1a, Supplementary Video S1). If the designated table is unavailable, the inhaler may be securely connected to the edge of the operation surface using tapes.

#### 2.2.2. Bent Needle

For both CSF collection and injection, insulin syringes (B.BRAUN, Melsungen, Germany, Omnican® 40, 30 G × 5/16”, 0.3 mm × 8 mm, 21B22CB) were used. To make the bent needle, the needle of a 30 G insulin syringe was taken off and the tip of the needle was held with hemostat forceps for approximately 2–3 mm for mice and 4–5 mm for rats. The tip was then bent to a 90–110° angle using the same forceps (Figure 1b).

#### 2.2.3. Collection Needle Set 

The intravenous infusion set (Shinva Shandong Medical Instrument Co., Ltd., Zibo, China, size 0.7, serial number 100019217960) was used to cut the infusion needle, leaving only the tubing with the Luer connector. The tubing was then connected to the needle of the 30 G insulin syringe (B.BRAUN, Melsungen, Germany, Omnican® 40, 30 G × 5/16”, 0.3 mm × 8 mm, 21B22CB) that had been bent as described earlier (Figure 1c). A portable holder was used to secure a 1-mL disposable, sterile syringe (Jiangsu Zhiyu Medical Instrument Co., Ltd., Taixing, China, 1 mL, 0.45 mm × 15 mm) (#1), which was then connected to the Luer connector of the previously assembled bent needle set (Figure 1d). An extra 1-mL syringe without a needle (#2) and a microcentrifuge tube were also prepared for CSF collection.

#### 2.2.4. Injection Needle Set

The injection needle set was assembled by inserting the bent 30 G insulin syringe needle (described in Section 2.2.2) about 5 mm deep into a sterilized PTFE tube (PURESHI, Shanghai, China, 0.7 × 1.7 mm) (Figure 1e). The length of the PTFE tube can be adjusted as needed. A 10-µL pipette tip was inserted into the other end of the PTFE capillary tube and tightly secured to a pipette (Figure 1e). The pipette was used to precisely control the volume of the injected solution.

### 2.3. Procedures of Animal Experiments

#### 2.3.1. Anesthetize Animals

The animal was first anesthetized with 3% isoflurane (RWD Life Science Co., Ltd., Shenzhen, China, R510-22) in the induction chamber. The animal was then checked for full anesthesia using the toe-pinching reflex method. Next, the head of the properly anesthetized animal was secured onto the custom-made surgical table, which was connected to an inhaled gas breathing tube. The nose and mouth of the animal were immediately placed into the facemasks and nosecones to maintain the anesthesia. To prevent ocular damage during the surgery, the eyes were optionally treated with erythromycin eye ointment (Beijing Shuangji Pharmaceutical Co., Ltd., Beijing, China, H11021270, 0.5%).

#### 2.3.2. CSF Collection

**Figure 2:**
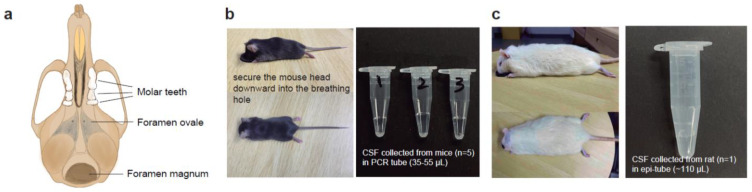
Graphical illustration and pictures for CSF collection in mice and rats. (**a**) Graphical scheme showing the basal view of a mouse skull, the foramen magnum is where the bent needle tip will be inserted for CSF collection in our method. (**b**) Image showing the position of the head of a laboratory mouse under anesthesia and during the CSF collection surgery. On the right, a photograph showing the CSF liquid collected from mice. Each tube contains the CSF merged from five mice, with the estimated liquid volumes (µL) indicated. (**c**) Image showing the head position of an experimental rat under anesthesia and during the CSF collection surgery. On the right, a photograph showing the CSF liquid collected from one rat with the estimated liquid volume indicated.

To collect CSF from rodents, the animal’s head was positioned vertically downward to locate the foramen magnum (Figure 2a–c, Supplementary Video S1). Once confirmed, the rodent’s head was sanitized with 75% ethanol (*vol*/*vol*). To prevent potential contamination during CSF collection, hair removal prior to the operation was recommended for both mice and rats. The bent needle was punctured vertically into the skin at the back of the foramen magnum while holding the mouse’s head gently and vertically downward with one hand. The bent needle was stably punctured into the skin. The plunger of syringe #1 was slowly pulled to draw the clear CSF into the tubing. The tubing was firstly detached from syringe #1 when the CSF flowed into it, then bent needle was gently removed from the foramen magnum using the other hand. The bent needle was transferred into a collection tube, and the Luer connector of the tubing was attached to syringe #2. The plunger was pushed, and the CSF was allowed to flow into the microcentrifuge tube. Approximately 5–10 µL and 70–100 µL of the CSF can be obtained from each mouse and rat, respectively, in a one-time collection (Figure 2b,c). Occasionally, we can obtain up to 15 µL of CSF from an adult mouse and up to 120 µL from an adult rat. The samples can be used for analysis immediately, or stored in −80 °C freezers or liquid nitrogen for future analysis. For operating on a rat, the anesthesia-breathable hole is larger than that of a mouse (Supplementary Video S2). Therefore, it is recommended to prepare a separate connecting tube with a larger breathing hole for the anesthesia equipment and the custom-made surgical table (Figure 1a).

#### 2.3.3. CSF Injection

A liquid transfer pipette was connected to the injection bent needle set and set to the injection volume calculated in advance. The desired solution was aspirated into the PTFE capillary tube by immersing the bent needle connected to the tubing into the solution and aspirating the desired volume of solution. Confirmation of the injection site was performed by confirming and feeling the location of the foramen magnum with a finger. Before puncturing the needle, the rodent’s head was sanitized with 75% ethanol (*vol/vol*). The bent needle is then punctured vertically into the skin at the back of the foramen magnum while holding the mouse’s head in position with one hand. When the bent needle punctures through the foramen magnum and is inserted into the cerebellomedullary cistern, the CSF flows into the PTFE capillary tube causing the fluid level to rise. The hand holding the animal can then be slowly released, and the pipette can be gently pressed. The solution inside the PTFE capillary tube should be injected into the CSF circulation by slowly pushing the pipette.

#### 2.3.4. Postoperative Care of the Animal

After the surgery for the collection or the injection from the anesthetized animal, the animal is placed on a 37 °C heating pad (KEWBASIS Nanjing, KW-XMT7100, Nanjing, China). Typically, the animal wakes up within 5–10 min and moves freely in a short period of time. Depending on the experimental needs, postoperative mice can be either used for surgical analysis and euthanization or transferred back to the nursing cage. For nursing, monitoring the health condition and behavior of the animal on a daily basis for at least 7 days is recommended. When anorexia, weakness, weight loss, or infection of body organs in the animal occurs, the animal should be cared for according to the animal welfare guidelines or euthanized.

### 2.4. Proteomic Analysis of Mouse CSF Samples

#### 2.4.1. Sample Preparation

We transferred 10 µL of each cerebrospinal fluid sample to a 1.5 mL centrifuge tube and lysed it with 50 µL of a lysis buffer including 8 M urea (Sinopharm Chemical Reagent Co., Ltd., Shanghai, China, AR-10023218), 100 mM triethyl ammonium bicarbonate (TEAB) (Sigma-Aldrich, St. Louis, MO, USA, T7408-500ML), pH 8.5. The lysate was centrifuged at 12,000× *g* for 15 min at 4 °C, and the supernatant was reduced with 10 mM DTT (Sigma-Aldrich, St. Louis, MO, USA, D9163-25G) for 1 h at 56 °C. The samples were subsequently alkylated with sufficient iodoacetamide (IAM) (Sigma-Aldrich, St. Louis, MO, USA, I6125-25G) for 1 h at room temperature in the dark.

#### 2.4.2. Trypsin Digestion

The volume of each protein sample was made up to 100 μL with a lysis buffer (8 M urea, 100 mM TEAB, pH 8.5), trypsin (Promega, Madison, WI, USA, V5280) and 100 mM TEAB buffer. Then, the samples were mixed and digested at 37 °C for 4 h. Then the trypsin was replenished and CaCl_2_ was added to digest overnight. Formic acid (Thermo Fisher Scientific, Waltham, MA, USA, A117-50) was mixed with a digested sample, the pH was adjusted to under 3, and centrifuged at 12,000× *g* for 5 min at room temperature. The supernatant was slowly loaded to a C18 desalting column (Waters, Milford, MA, USA, BEH C18, 4.6 × 250 mm, 5 μm), washed with a washing buffer containing 0.1% formic acid, 3% acetonitrile (Thermo Fisher Scientific, Waltham, MA, USA, A955-4) thrice, then an elution buffer comprising 0.1% formic acid and 70% acetonitrile was added. The eluents of each sample were collected and lyophilized.

#### 2.4.3. Separation of Fractions

Mobile phases A (2% acetonitrile, pH adjusted to 10.0 using ammonium hydroxide) and B (98% acetonitrile, pH adjusted to 10.0 using ammonium hydroxide) were used to develop the gradient elution. The lyophilized powder was dissolved in solution A and centrifuged at 12,000× *g* for 10 min at room temperature. The sample was fractionated using a C18 column (Waters, Milford, MA, USA, BEH C18, 4.6 × 250 mm, 5 μm) on a Rigol L3000 HPLC system; and the column oven temperature was set to 45 °C. The detail of the elution gradient is shown in Table 1. The eluates were monitored at UV 214 nm, collected for a tube per minute and combined into 10 fractions. All the fractions were dried in a vacuum, and then, reconstituted in 0.1% (*v*/*v*) formic acid (FA) in water.

#### 2.4.4. LC-MS/MS Analysis

Mobile phase A (100% water, 0.1% formic acid) and B solutions (100% acetonitrile, 0.1% formic acid) were prepared. The lyophilized powder was dissolved in 10 μL of solution A, centrifuged at 14,000× *g* for 20 min at 4 °C and 200 ng of the supernatant was injected into the Liquid chromatography-mass spectrometry system for detection. The model type of the UHPLC was nano-upgraded nanoElute, and the analytical column was a homemade analytical column (15 cm × 100 μm, 1.9 μm). The elution conditions of liquid chromatography consist of using a 90-min elution gradient method, with a flow rate of 300 (mL/min), 5% mobile phase A and 95 % mobile phase B. Mass spectrometry analysis was performed on a Tims-TOF-Pro2 mass analyzer (Bruker Daltonics) with a captive spray ion source. The spray voltage was set to 2.1 kV. The full scan range of the mass was from *m*/*z* 100 to 1700 and the ramp time was 100 ms. The lock duty cycle was set to 100%. The settings of PASEF were as follows: 10 MS/MS scans (a total cycle time of 1.17 sec), ionic strength threshold of 2500 and scheduling target intensity of 20,000. The raw data of MS detection was named “.d”.

#### 2.4.5. Data Analysis

The resulting spectra were sought against the UniProt mouse proteome database by the MaxQuant (Bruker Daltonics, https://www.bruker.com/en/products-and-solutions/mass-spectrometry/timstof/timstof-pro-2.html, accessed on 5 May 2023) search engines. The search parameters of MaxQuant were set as follows: mass tolerance for the precursor ion was 20 ppm and the mass tolerance for the ion product was 0.05 Da. Carbamidomethyl was specified as fixed modifications, oxidation of methionine (M) was specified as dynamic modification, and acetylation was specified as N-terminal modification. A maximum of two missed cleavage sites were allowed. Retrieved results were further filtered in PD or MaxQuant softwares: peptide spectrum matches (PSMs) with a credibility of more than 99% were identified as PSMs. The identified protein contains at least one unique peptide. The identified PSMs and protein were retained and formed no more than 1.0% with FDR.

### 2.5. Reagents for Injection

Evans blue powder (Coolaber, Beijing, China, CE5171) was prepared as 10 mg/mL stock solutions in sterile saline. Hoechst 33342 (Beyotime Biotechnology, Shanghai, China, 10 mg, C1022) was dissolved in distilled water at a concentration of 10 mg/mL. The dissolved Hoechst 33342 stock solution was used for CSF injection directly, and the injection volume was calculated based on the animal body weight. For example, for a 7-month old C57BL/6 mouse weighing 45 g, 4 μL of stock solution was injected for each mouse.

### 2.6. Microscope Imaging

After the Hoechst 33342 was injected into the mouse CSF circulation, the whole brain organ was surgically dissected 1 h after surgery and then fixed in a 4% paraformaldehyde fix solution (Beyotime, P0099-500 mL) for 4 h. Subsequently, the brain was dehydrated in a 30% sucrose solution (Sigma-Aldrich, St. Louis, MO, USA, V900116-500g) dissolved in PBS overnight, and cryo-sectioned at 10 µm sagittally on an adhesion microscope slide using a Leica CM1950 cryostat. The sectioned slides were then covered with a microscope cover slide and antifade mounting medium (Beyotime, P0126-5 mL). Fluorescent images were taken using a Keyence BZ-X Series fluorescence microscope (Keyence Corporation, Osaka, Japan).

## 3. Results

### 3.1. Non-Invasive CSF Collection in Laboratory Rodents

Collecting CSF from small animals, especially mice, has been technically challenging or laborious due to the sophisticated surgical procedures and high mortality rates. Using our invented method, we can consistently collect 5–10 µL of CSF from adult mice and 70–100 µL from adult rats (Figure 2b,c). To ensure that our method does not pose significant adverse effects to the animal’s health, we monitored the recovery rate and survival rate of postoperative mice. In our CSF collection experiment, mice typically recover from anesthesia within 1–5 min without notable health issues, supporting the utility of this technique for studies requiring rapid post-surgery recovery in laboratory animals. 

**Figure 3:**
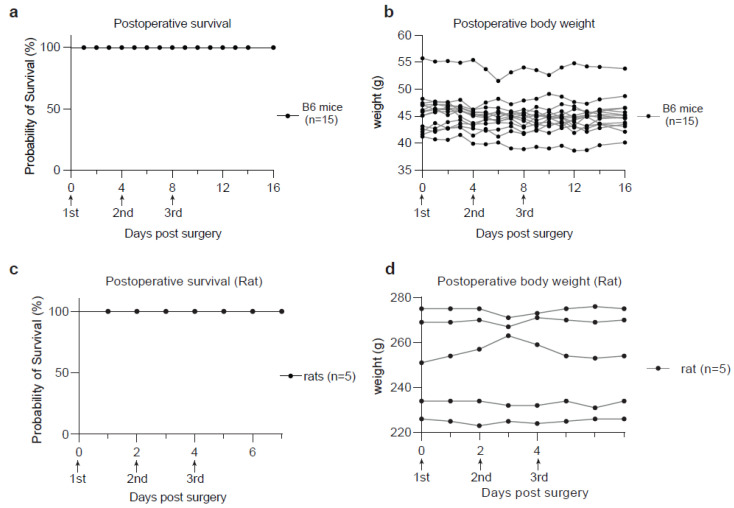
Animal survival and body weight monitoring after CSF collection. (**a**) Kaplan-Meier curve showing the survival rate of postoperative B6 mice (n = 15). (**b**) Curves showing the time-course monitoring of body weights in the postoperative B6 mice used in (**a**). (**c**) Kaplan-Meier curve showing the survival rate of postoperative rats (n = 5). (**d**) Curves showing the time-course monitoring of body weights in the postoperative rats used in (**c**).

Encouraged by the rapid postoperative recovery of the rodents, we attempted to collect CSF repetitively from the same animal using our method after sufficient recovery time. The experiment involved three rounds of CSF collection in a group of 15 adult mice on day 0, 4 and 8, respectively. All collections were successful without inducing significant changes in body weight or other health parameters (Figure 3a,b). The volumes of CSF extracted in later collections were comparable to those in the first collection. These mice survived another 10-day monitoring period without showing any notable signs of distress (Figure 3a,b). Similarly, the rats used in the CSF collection also demonstrated a rapid postoperative recovery and good survival from sequential CSF extraction on day 0, 2 and 4, respectively (Figure 3c,d). These results indicate that our method is compatible with longitudinal studies requiring serial CSF collection from the same animal, a major advantage over prior protocols that require extensive surgical operations.

### 3.2. Validation of the Content in the Isolated CSF Solutions

**Figure 4:**
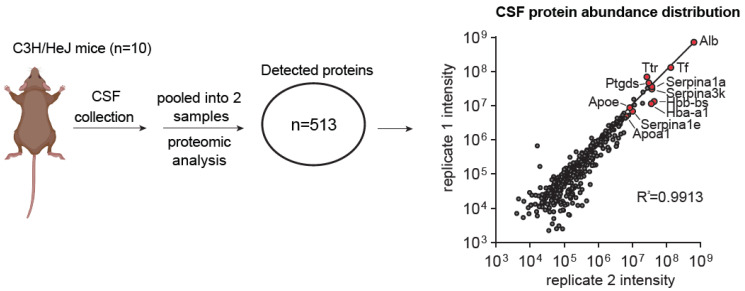
Proteomic analysis of mouse CSF collected using the bent needle method. Graphical scheme and scatter plot describing the proteomic analysis of CSF collected from adult mice and the CSF proteome abundances from two replicate pooled samples. Each sample contains the CSF pooled from 5 mice. Highly abundant proteins detected in both samples are selectively labeled in red. Correlation analysis is performed using the Pearson correlation method.

To validate our technique, we performed proteomic analysis to identify previously characterized molecular markers in the isolated CSF samples (Figure 4a). Mass spectrometry analysis detected at least 513 proteins in the pooled mouse CSF samples (Figure 4a, Supplementary Dataset S1). These proteins include Apolipoprotein E (Apoe) and Apolipoprotein A-1 (Apoa1), which are the predominant apolipoproteins in mammalian CSF [24], as well as Amyloid β precursor protein (App) [25], Serotransferrin (Tf) [26], Transthyretin (Ttr) [26], Prostaglandin D2 Synthase (Ptgds) [26], etc. (Figure 4a). These molecular features indicate that we were successfully collecting the cerebrospinal fluid from the mouse.

### 3.3. Confirmation of Delivering Liquid Reagent into CSF Circulation

**Figure 5:**
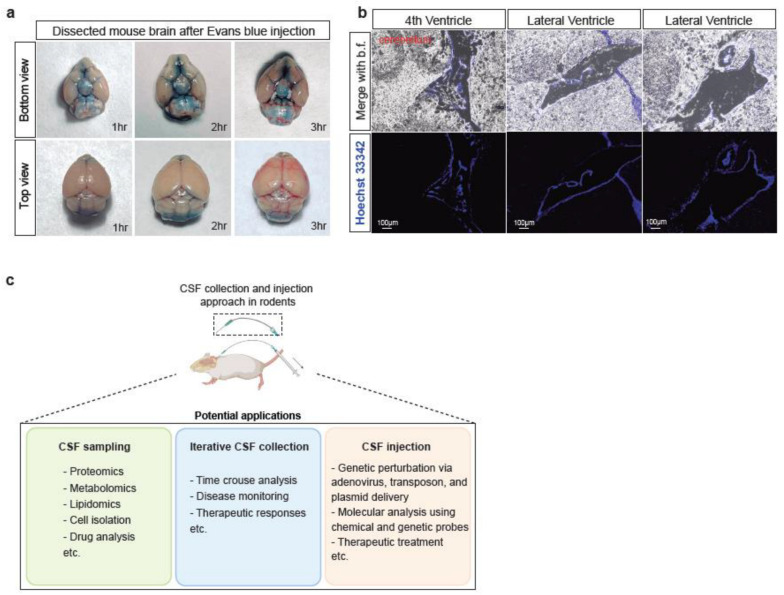
Liquid reagents can be delivered to the mouse CSF circulation via the bent needle method. (**a**) Optical imaging of the dissected mouse brains 1-, 2-, and 3- h after injection of 1% Evans blue solution. The brain images are depicted in both top and bottom views. (**b**) Fluorescence microscopic images of mouse brain in sagittal plane after injecting Hoechst-33342 (blue) for an hour. Scale bar is 100 μm. (**c**) Scheme showing the proposed utilities of the developed CSF collection and infusion methods.

To confirm the efficacy of our CSF injection method, we first used Evans blue, a metabolically inert dye, as a coloured marker to track the flow of injected solutions. Upon dissecting the mice’s brains 1-, 2- or 3-h post-injection, we observed that the dye was distributed on a wide range of brain surfaces, including areas that were distant from the injection site at the foramen magnum. This diffusion pattern was consistent with the coverage of the CSF circulation on the rodent brain surface (Figure 5a). These results demonstrate the success of our bent needle injection method in delivering liquid reagents on a lower µL scale to the CSF circulation of the mouse.

### 3.4. Confirmation of Delivering Liquid Reagent into Brain Ventricles

In addition to the successful confirmation of CSF injection using the Evans blue dye, we also injected Hoechst 33342 into the CSF circulation to evaluate the efficacy of our method in delivering liquid reagents inside the brain. Hoechst 33342 is a fluorescent dye commonly used to stain DNA in live cells with low cytotoxicity. In our study, we found that Hoechst 33342 injected via our minimally-invasive CSF injection method could reach and circulate in the brain ventricles, including the fourth ventricle and the lateral ventricles (Figure 5b). The ventricles are filled with CSF due to it being a system of four interconnected fluid-filled spaces within the brain [27]. This result suggests that our method is not only effective in delivering reagents into the CSF circulation but also allows for their distribution to the brain ventricles. This characteristic supports the possible use of our method for studying various brain regions that are in direct contact with CSF, such as the choroid plexus, a highly vascularized tissue responsible for producing the CSF in the brain [28,29].

## 4. Discussion

The role of the CSF in the central nervous system (CNS) includes maintaining homeostasis by supplying nutrients, removing waste products and protecting against mechanical insults [30,31]. The CSF also provides an important window for evaluating the physiological and pathological states of the CNS. However, a key bottleneck in the currently existing CSF collection and infusion methods is the requirement of the surgical opening of the mouse head/neck skin to help the operator visualize the liquid chamber in the CNS and prevent the over-puncturing of solid tissues by the metal needles or glass capillaries. The heavy reliance on extensive experiences and practices limits the widespread use of such methods by experimental scientists.

To overcome this issue, we introduce a new approach utilizing a unique bent needle set that connects to a pipette, allowing for precise and efficient CSF collection and injection. In this method, we created a set of bent metal needles that are specifically designed to meet the required distances between the skin and foramen magnum near the back of the neck of the experimental mice and rats. This tool constitutes the major feature of our protocol. Using this bent needle, our method does not rely on the operator’s skill to discern the changes in the resistance during the needle’s penetration into the CSF liquid space and transition towards solid tissues. Notably, by eliminating the necessity of visualizing the CSF liquid chamber by the operator directly, our approach bypasses the surgical opening of the rodent’s head/neck skin, which is laborious, invasive and potentially detrimental. With sufficient practice, the total time required for collecting the CSF from each mouse can be as short as 2–5 min, which significantly simplifies the procedure and reduces the animal’s distress. Using this method, we were able to obtain the CSF from adult mice and rats without the need to sacrifice the animal, yielding 5–10 µL and 70–100 µL per procedure, respectively. These volumes are comparable with those from most other methods. Through this approach, the collected CSF samples are compatible with various downstream analyses, including biochemical assays and metabolomic, lipidomic and proteomic analyses.

Inspired by the minimal invasiveness and high postoperative animal survival rates of our CSF collection approach, we adapted the same bent needle tools for CSF injection via the foramen magnum. In this part of the method, we inserted an elastic polytetrafluoroethylene (PTFE) capillary tube into a 30 G syringe bent needle to make the injection needle set. Once the connection was tightly contacted, we secured a pipette with a tip to the bent needle set then the injected amount could be precisely controlled. We injected 5 µL of the desired solution such as the Evans blue dye and Hoechst 33342 into the CSF of the mouse. By dissecting mice at different time points after injection, we confirmed that the Evans blue dye and the Hoechst 33342 solutions were successfully injected into the mouse’s CSF circulation and circulated into the brain ventricles. Notably, the ease of operation provided by the bent needle tool helped us to shorten the full injection procedure to 3–5 min, a significant improvement over the prior methods. Moreover, the reduced invasiveness of the protocol allowed the animals to return to normal activity within 5–10 min, while none of the animals required euthanization. We envision our easy-to-practice and cost-effective method will be broadly useful for accessing the CSF in experimental animal models.

One limitation of our protocol is that the operator needs to precisely locate the foramen magnum. In our experience, to locate the foramen magnum structure accurately, the animal’s head should be placed vertically downward. The experimental operator should use one hand to hold the neck of the animal and gently push the head forward, while using the other hand to feel the location of the foramen magnum, until finding a favorable position to carry out the surgery. Furthermore, we suggest using proper aseptic tools and handling the animals in a sterile environment during the surgery to minimize the risk of infection.

By introducing the key bent needle tool described above, we envision that the minimally-invasive CSF collection and infusion methods presented here will enable more efficient monitoring of the functional states of the central nervous system in research involving small laboratory animals, including mice and rats. This approach allows multi-omic profiling of the collected CSF, including proteomics, metabolomics and lipidomics, as well as cell isolation for characterization of the CSF compositions (Figure 5c). Notably, iterative CSF collection from the same animal will enable new experimental strategies that were previously not possible or too laborious and costly, including time-course tracking of disease progression and therapeutic responses. Moreover, our method is compatible with delivering various reagents into the CSF, including plasmids, chemical probes, proteins, peptides and adenoviruses, which will likely improve the technical efficiency in CNS disease modeling, tracing and monitoring CNS functions, and imposing therapeutic treatments. We envision that our technique will be widely useful at the forefronts of neuroscience.

## 5. Conclusions

In conclusion, the CSF-accessing method using the bent needle tool introduced in this study is minimally-invasive, cost-effective, and allows precise and efficient collection and injection of CSF in different-aged mice and rats. This method eliminates the need for surgically opening the skin above the rodent’s head or neck, significantly simplifying the procedure and reducing the animal’s distress.

Using this approach, CSF samples can be collected from the same animal repetitively, enabling new experimental strategies such as the time-course monitoring of the disease progression and therapeutic responses. The collected CSF samples are compatible with various downstream analyses, including proteomics, metabolomics and lipidomics. Also, various reagents can be delivered to the same animal at serial times, making drug testing or model construction much easier and more conceivable. Collectively, this approach offers an effective and low-cost alternative to traditional methods, providing researchers with a more accessible and precise way to collect and analyze CSF samples for advancing neuroscience research.

## Figures and Tables

**Table 1:** Nanoelute liquid chromatography elution gradient table.

Time (min)	Flow Rate (mL/min)	Mobile Phase A (%)	Mobile Phase B (%)
0	300	98	2
45	300	78	22
50	300	65	35
55	300	20	80
60	300	20	80
75	300	78	22
80	300	63	37
85	300	5	95
90	300	5	95

## Data Availability

Data supporting the findings of this study are available in this article and/or in Supplementary Materials.

## Supplementary Materials

The following supporting information can be downloaded at: https://www.mdpi.com/article/10.3390/biomedicines11061609/s1, Video S1. Experimental procedure for CSF extraction from a mouse; Video S2. Experimental procedure for CSF extraction from a rat. Dataset S1. Proteomic analysis of mouse CSF samples.
